# An improved method for the incorporation of fluoromethyl ketones into solid phase peptide synthesis techniques†

**DOI:** 10.1039/d1ra03046a

**Published:** 2021-06-08

**Authors:** Dhira Joshi, Jennifer C. Milligan, Theresa U. Zeisner, Nicola O'Reilly, John F. X. Diffley, George Papageorgiou

**Affiliations:** Peptide Chemistry STP, The Francis Crick Institute 1 Midland Road London NW1 1AT UK George.Papageorgiou@crick.ac.uk +44 (0)203 796 2359; Chromosome Replication Laboratory, The Francis Crick Institute 1 Midland Road London NW1 1AT UK; Cell Cycle Laboratory, The Francis Crick Institute 1 Midland Road London NW1 1AT UK

## Abstract

An improved and expedient technique for the synthesis of peptidyl-fluoromethyl ketones is described. The methodology is based on prior coupling of an aspartate fluoromethyl ketone to a linker and mounting it onto resin-bound methylbenzhydrylamine hydrochloride. Subsequently, by utilising standard Fmoc peptide procedures, a number of short Z-protected peptides were synthesised and assessed as possible inhibitors of the main protease from SARS-CoV-2 (3CL^pro^).

## Abstract 2

An improved and expedient technique for the synthesis of peptidyl-fluoromethyl ketones is described.

## Graphical abstract

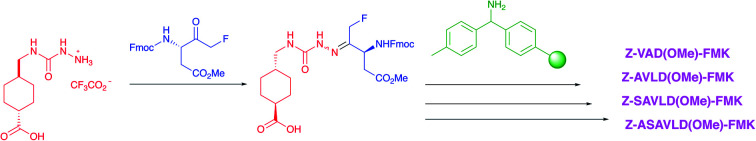

## Introduction

Although peptidyl-fluoromethyl ketones (PFMKs) were postulated to be effective serine protease inhibitors in the late 1960s,^1^ their efficient and atom economic synthesis still remains a key objective for peptide chemists. The first syntheses of fluoromethyl ketones (FMKs) were independently reported by the Rasnick,^2^ Shaw^3^ and Imperiali^4^ groups in the mid-1980s. Since then, numerous reported syntheses of PFMKs have appeared in the literature together with comprehensive reviews^1,5–8^ describing their widespread use as probes for serine and cysteine proteases and as potential drugs for rheumatoid arthritis^9,10^ and other diseases.^11,12^

PFMKs are well known as inhibitors of serine and cysteine proteases, such as caspases, cathepsins and Sentrin/SUMO specific proteases.^13–15^ Their peptide backbone can be altered to mimic a substrate sequence that binds directly to the active site of a protease. This makes PFMKs excellent tools for target-based inhibition of specific proteases. The 3C-like protease (3CL^pro^)^16^ of severe acute respiratory syndrome coronavirus 2 (SARS-CoV-2), the causative agent of the global COVID-19 pandemic, is a particularly interesting therapeutic target. The SARS-CoV2 genome encodes two overlapping polyproteins, from which the functional polypeptides have to be released principally by proteolytic processing by 3CL^pro^.^17,18^ Therefore, this cysteine protease is essential for the viral replicative cycle. Human proteases with similar cleavage specificity are very rare, making it an attractive target for inhibitor design.^19^ Its substrate sequence preference is known, and thus it is a good candidate to demonstrate the effectiveness and specificity of short PFMK inhibitors. Since the nsp4/5 cut site is predicted to have the highest affinity for 3CL^pro^, the peptidyl moiety was based on this sequence, except for an aspartic acid instead of a glutamine at the P1 site due to ease of synthesis.^20^

PFMKs are usually obtained by incorporating the corresponding fluoroalcohol into a peptide sequence, by a solution coupling with the carboxylic acid terminus of a peptide, followed by alcohol oxidation.^4,21–25^ However, this methodology is limited to peptide sequences that either do not contain cross-reacting sidechain groups, have their sidechains orthogonally protected, or do not contain oxidation sensitive residues such as Cys, Met, Trp or Tyr. Furthermore, the solubility issue associated with fully protected amino acids in solid phase peptide synthesis (SPPS) and the racemisation possibilities at the C-terminal amino acid limit the capacity of this synthetic procedure. Therefore, there is scope for prior formation of the amino acid FMK moieties which can be incorporated to a peptide sequence either by solution chemistry or by SPPS techniques.

## Results and discussion

The most commonly reported methodology for the synthesis of N-protected amino acid FMKs is based on the conversion of halomethyl ketones (mostly bromo or chloro) to the corresponding fluoro analogues. Halomethyl ketones are first prepared by reacting the mixed anhydride or otherwise activated acid with diazomethane, to give the corresponding diazoketone which upon treatment with anhydrous HCl or aqueous HBr affords the required halomethyl ketone. Reeder and Anderson's^26^ comprehensive review listed the most common reagents and conditions for these transformations. Alternative procedures, which avoid the use of diazomethane, also require hazardous reagents and conditions. Diazomethane still remains the most effective reagent for this transformation despite associated safety concerns due to its toxicity, thermal instability and shock sensitivity, especially in large scale reactions. TMS–CHN_2_ is considerably less toxic than diazomethane itself, but it has been reported^27^ that it does not react with mixed anhydrides derived from amino acids, possibly due to steric hindrance.

As a part of our aim to synthesise a number of short Z-protected peptidyl-FMKs we initially based our investigations on a published procedure^28^ which claimed the synthesis of fluoromethyl dimethylketal **1**. We envisioned to use this reagent in SPPS procedures to construct the desired short peptides. We present here the shortcomings encountered with this approach and report an alternative new viable methodology for the synthesis of PFMKs.
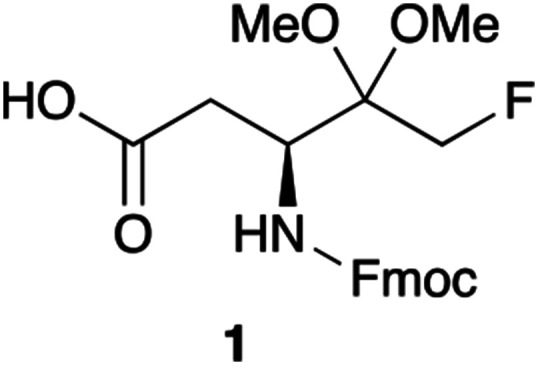


**Scheme 1:**
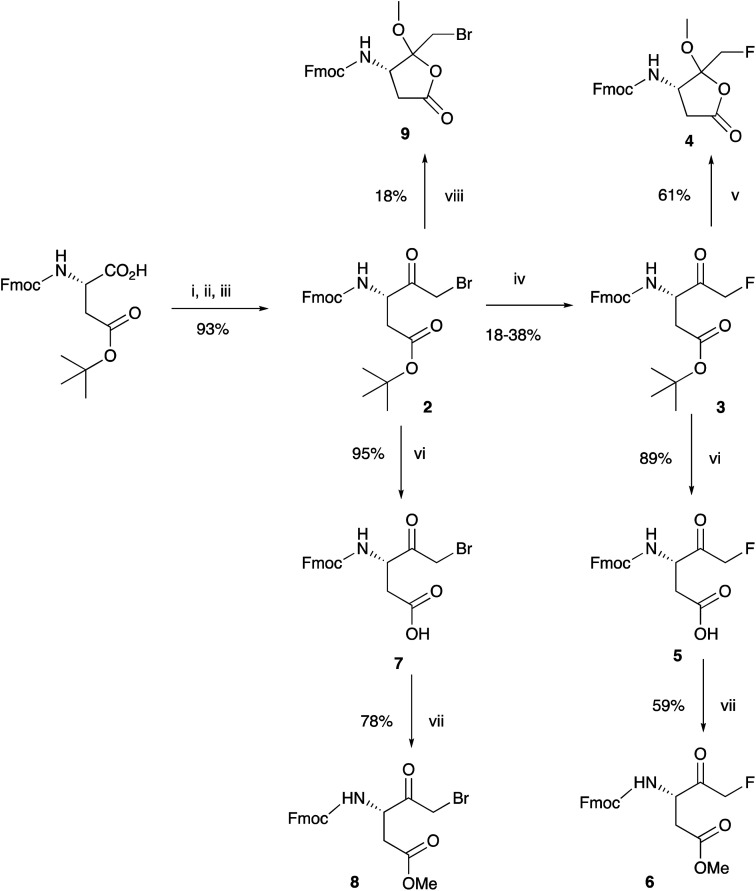
Synthesis of key synthon **6**. Reagents and conditions: (i) i*so*-butyl chloroformate, NMM, THF, −10 °C; (ii) CH_2_N_2_, Et_2_O, 0 °C to rt; (iii) 33% HBr in AcOH–H_2_O (1 : 2); (iv) TBAF, *p*-TsOH, THF, reflux; (v) *p*-TsOH, MeOH, toluene, 60 °C; (vi) CH_2_Cl_2_, TFA, 0 °C; (vii) Me_3_SiCl, MeOH; (viii) 2,2-dimethoxypropane, MeOH, *p*-TsOH, 60 °C.

Aspartic acid fluoromethyl ketone (D-FMK) is an important synthon for the construction of highly desirable short peptides such as Z-VAD-(OMe)-FMK, which is used as a caspase inhibitor.^29,30^ Our initial strategy was to prepare compound **1** in a useful quantity and use it as the first amino acid unit in standard Fmoc-SPPS protocols. The aim was to attach it to the resin, construct the required peptide and methylate the acid side group after cleavage from the solid support. As depicted in Scheme 1, starting from the commercially available Fmoc-Asp-(O^*t*^Bu)-OH we prepared the bromomethyl ketone **2** in multigram quantity in an excellent yield in a tandem reaction manner without isolating the intermediate diazoketone derivative. Although the conversion of **2** to the fluoromethyl ketone **3**, was reported^28^ at 49% yield, in our hands gave variable 18–38% yields. A brief effort to improve the yield of this reaction using a variety of fluoride sources such as KF, LiF, or TBAF under various conditions (data not shown) resulted either in complete cleavage of the Fmoc group or no reaction. With fluoromethyl ketone **3** in hand we proceeded to protect the ketone functionality as a dimethylketal, as described^28^ with the objective of obtaining compound **1**. To our initial surprise the only isolated product proved to be the lactone **4**. The structure of the product was confirmed by both 1-dimensional high field ^1^H, ^13^C and ^19^F NMR spectroscopy and also by ^1^H–^1^H COSY and ^1^H–^13^C HSQC, correlation spectroscopy (see ESI†). Halomethyl ketones **3** and **2** were easily transformed to the corresponding methyl esters **6** and **8** respectively, either by isolating the corresponding acids **5** and **7**, or by *in situ* deprotection with TFA and methylation with TMS–Cl in MeOH. The use of these reagents in this study is discussed below.

**Scheme 2:**
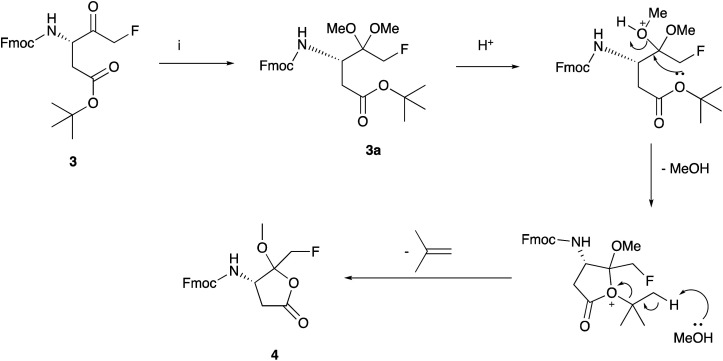
Proposed mechanism for the formation of **4**. Reagents and conditions: 10% *p*-TsOH, MeOH, 60 °C.

In order to further investigate this unexpected cyclisation, we attempted ketone protection on the more readily available bromomethyl ketone **2** under a variety of appropriate conditions, using either methanol or 2,2-dimethoxypropane for ketal formation. In all cases, the isolated product was the lactone **9**. We also found that attempting to form a cyclic ketal using ethylene glycol predominantly gave a lactone (data not shown). We therefore concluded that the intermediate dimethyl ketal **3a** (Scheme 2) generated from fluoromethyl ketone **3**, is unstable under the mild acidic conditions. Cyclisation with concomitant loss of methanol and isobutylene generates the thermodynamically stable lactone **4**. Our proposed mechanism is depicted in Scheme 2. Despite using similar experimental conditions Funeriu *et al.*,^28^ did not observe the formation of **4** and reported the isolation of **1** in 77% yield. Nonetheless, they did not report its incorporation into peptide sequence either by solution chemistry or by SPPS methods. In fact, they protected the ketone function of Fmoc-glycidyl-FMK as a cyclic acetal which was then attached to a linker *via* a click chemistry reaction.

**Scheme 3:**
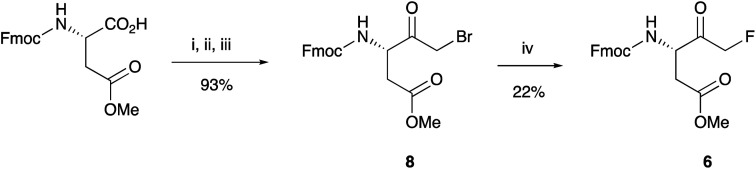
Shorter synthesis of key synthon **6**. Reagents and conditions: (i) *iso*-butyl chloroformate, NEM, THF, −10 °C; (ii) CH_2_N_2_, Et_2_O, 0 °C to rt; (iii) 33% HBr in AcOH–H_2_O (1 : 2); (iv) TBAF, *p*-TsOH, THF, reflux.

The failure to prepare the desired FMK **1** prompted us to seek an alternative approach. Since the protection of the ketone functionality as a ketal proved problematic, we decided to use Fmoc-Asp(OMe)-FMK as our key synthon, which enabled shorter synthesis and atom economy from commercially available Fmoc-Asp(OMe)-OH. Thus, bromomethyl ketone **8** was obtained in an excellent yield using the same one-pot procedure described above. Although conversion to FMK **6** was accomplished in relatively low yield, owing to the issues described above, the shorter synthetic pathway compensates for this shortcoming (Scheme 3).

**Scheme 4:**
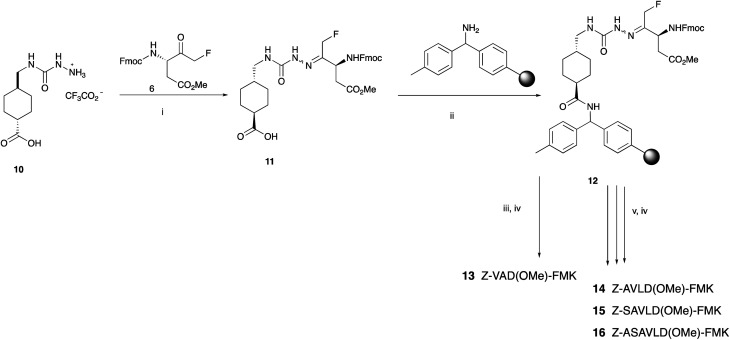
Synthesis of FMK-peptides **13–16**. Reagents and conditions: (i) NaOAc, EtOH, H_2_O, reflux; (ii) HATU, DMF, DIPEA; (iii) non-automated SPPS using Fmoc aa, HATU, DIPEA, DMF; (iv) 95% TFA, 2.5% H_2_O, 2.5% TIPS; (v) fully automated SPPS using Fmoc aa, HATU, DIPEA, DMF.

It was then envisaged that the best way forward was to reversibly anchor the ketone to an easily accessible linker which could be attached to an appropriately functionalised resin. The 1,3-diol linker described by Funeriu *et al.*^28^ was not an attractive option because of the extensive synthetic work required, and the harsh conditions (37% HCl) needed for cleavage. Quibell and co-workers^31^ reported the condensation of bicyclic ketones with the hydrazide linker **10** described by Murphy *et al.*^32^ This was considered more suitable due to its robustness and straightforward short synthesis. Also, the ease of cleavage using relatively mild conditions made this method more attractive. Thus, the linker **10** was prepared in multigram quantity from commercially available tranexamic acid in an excellent overall yield (see ESI†). Condensation of FMK **6** with **10** gave the desired hydrazone **11** in good yield. To confirm the viability of this approach, **11** was coupled to resin-bound 4-methylbenzhydrylamine (MBHA) hydrochloride to form **12**. We first prepared the commercially available Z-VAD(OMe)-FMK **13** using non-automated SPPS protocols. Subsequently with the effectiveness of the method established, we used fully automated SPPS protocols to synthesise a further three short peptides, Z-AVLD(OMe)-FMK **14**, Z-SAVLD(OMe)-FMK **15** and Z-ASAVLD(OMe)-FMK **16**. The purity of all peptides was estimated by integration of the peak from LCMS analysis (Scheme 4).

**Fig. 1:**
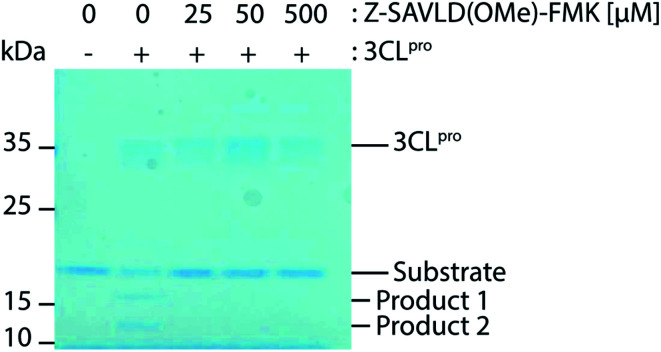
Gel based cleavage assay.

The usefulness of the synthesised short peptides **13–16** as inhibitors of 3CL^pro^ of SARS-CoV-2 was investigated and full disclosure of biological results will appear elsewhere.^33^ An example of the use of Z-SAVLD(OMe)-FMK **15** against the SARS-CoV-2 3CL^pro^ main protease is disclosed here. The *in vitro* inhibitory effect of the 3CL^pro^ specific PFMK was investigated using a gel-based protease assay. Briefly, a fusion protein was constructed from non-structural protein 9 (nsp9) of SARS-CoV-2 with an N-terminal FLAG-His epitope. A short linker sequence based on the natural nsp4/5 cleavage site connects the epitope and nsp9. Upon incubation with 3CL^pro^, the FLAG-His-tag is cleaved off from nsp9 at this cleavage site, resulting in two products visible at approximately 11 kDa and 16 kDa in the absence of the inhibitor (Fig. 1). In the presence of varying concentration of the PFMK inhibitors 3CL^pro^ protease activity is completely abolished, even at the lowest concentration tested (25 μM). This demonstrates that PFMKs might be a useful tool for probing 3CL^pro^ enzymatic activity. However, their development into clinical drugs is unlikely, due to their high *in vivo* toxicity, which might be explained by the metabolic conversion of the FMK group into toxic fluoroacetate.^34^

## Conclusions

In this paper we describe an improved general and efficient method for the preparation of PFMKs using standard Fmoc SPPS chemistry. This methodology permits the efficient introduction of aspartate fluoromethyl ketone at C-terminal end of any peptide sequence. It also demonstrates that conversion of the α-carboxyl group to fluoromethyl ketone functionality is feasible to most Fmoc protected amino acids prior to appropriately side chain group protection where is a prerequisite. The ease of condensation of the ketone function with the functionalised hydrazine linker allows the introduction of any amino acid as first unit in the sequence. Subsequent coupling to resin-bound MBHA followed by standard Fmoc-SPPS techniques will give access to the desired PFMK with any amino acid at the C-terminal. The usefulness of the technique is demonstrated by the synthesis of short peptides **13–16** and their application to current investigations for a therapeutic target of SARS-CoV-2.

## Experimental section

### General experimental information

Air or water sensitive reactions were performed in anhydrous solvents under a positive pressure of nitrogen. Dried and other solvents, reagents and resins were purchased from commercial suppliers and used without further purification. NMR spectra were recorded on Bruker Avance III spectrometers operating at 400, 600, 800 and 950 MHz in CDCl_3_ solution with TMS as internal reference, unless otherwise specified. Chemical shifts (*δ*) are reported in parts per million (ppm). Multiplicities are abbreviated as broad (br) singlet (s) doublet (d) triplet (t) quadruplet (q) and multiplet (m). Elemental analyses were carried out by MEDAC Ltd., Surrey, UK. Thin layer chromatography (TLC) was performed on Merck silica gel coated glass F_254_ plates and were visualized by UV irradiation at 254 nm. Preparative column chromatography was performed on Biotage Isolera One 3.3.0. All extracts of organic solvents were dried over anhydrous Na_2_SO_4_ and evaporated under reduced pressure.

Diazomethane generation should be carried out in a fast-flow ventilated fume cupboard using only glassware with Clear-Seal joints (no grinded joints) and without any means of stirring. All necessary safety precautions should be strictly applied! The generated amount of reagent should be used immediately avoiding any long-term storage.

For diazomethane generation and use in the experiment described below, a mixture of 2-(2-ethoxyethoxy) ethanol (84 mL) and ether (48 mL) was added to a solution of KOH (15 g) in water (24 mL) in a 500 mL round bottom flask attached to a condenser and receiving flask without stirring or edging. The reaction vessel was warmed to 65 °C on water-bath and a solution of Diazald (*N*-methyl-*N*-nitroso-*p*-toluenesulfonamide, 30 g) in diethyl ether (270 mL) was added slowly from a dropping funnel in similar rate to distillation. After the addition of all Diazald solution, more diethyl ether (60 mL) was added to the dropping funnel and distillation continued until the distillate became colourless. The distilled ethereal yellow solution of diazomethane was collected in a 500 mL conical flask which was pre-cooled to −70 °C in an acetone-dry ice bath containing KOH pellets. The generated diazomethane solution was then transferred *via* a cannula under nitrogen to the reaction vessel and used immediately.

#### *tert*-Butyl (*S*)-3-{[(9*H*-fluoren-9-yl)methoxycarbonyl]amino}-5-bromo-4-oxopentanoate **2**

To a solution of Fmoc-l-Asp(O^*t*^Bu)-OH (12.34 g, 30 mmol) in dry THF (150 mL), cooled to −10 °C was added *N*-methylmorpholine (NMM) (3.79 g, 37.5 mmol) and *iso*-butyl chloroformate (4.71 g, 34.5 mmol). A white precipitate was formed instantly, and the mixture was stirred at −10 °C for 1 h. Diazomethane (extra caution should be applied for the generation and handling of this reagent!), generated *in situ* using the procedure described in Aldrich Technical Bulletin (AL-180), (99.6–128.4 mmol), in Et_2_O dried over KOH pellets, was added slowly to the stirred reaction mixture at 0 °C. The solution was allowed to warm to rt overnight and then cooled to 0 °C and treated with a solution of 33% HBr in AcOH and water (1 : 2) (90 mL). The reaction mixture was first stirred at 0 °C for 1 h and then at rt for further 1 h until the evolution of gas ceased. The mixture was diluted with EtOAc (300 mL) and washed sequentially with water, saturated aq. NaHCO_3_ and brine. The organic phase was dried and evaporated to a pale viscous oil which crystallised after trituration with ether. Recrystallization afforded **2** (ref. 28) (13.61 g, 93%) as white crystals, mp 114–116 °C (EtOAc–hexanes). Anal. Calcd for C_24_H_26_NBrO_5_: C, 59.02; H, 5.37; N, 2.37. Found: C, 59.57; H, 5.24; N, 2.99%.

#### *tert*-Butyl (*S*)-3-{[(9*H*-fluoren-9-yl)methoxycarbonyl]amino}-5-fluoro-4-oxopentanoate **3**

To a solution of **2** (3.91 g, 8 mmol) and *p*-TsOH·H_2_O (4.59 g, 24 mmol) in THF (120 mL) was added dropwise a solution of TBAF·H_2_O (12.55 g, 48 mmol) in THF (120 mL) and the mixture was stirred in a nitrogen atmosphere under reflux overnight. After cooling to rt the solvent was evaporated, and the residue was dissolved in EtOAc (200 mL) and washed with water and brine. The organic phase was dried and evaporated, and the residue was flash chromatographed [EtOAc–hexanes (1 : 9) to (1 : 4)] to give **3** (ref. 28) (1.29 g, 38%) as pale crystals, mp 56–58 °C (Et_2_O–hexanes). Anal. Calcd for C_24_H_26_NFO_5_: C, 67.43; H, 6.13; N, 3.28. Found: C, 67.42; H, 6.09; N, 3.41%.

#### (9*H*-Fluoren-9-yl)methyl [(3*S*)-2-(fluoromethyl)-2-methoxy-5-oxotetrahydrofuran-3-yl]-carbamate **4**

To a solution **3** (0.92 g, 2.15 mmol) in toluene (92 mL) and MeOH (9.2 mL) was added *p*-TsOH·H_2_O (41 mg, 0.215 mmol) and the mixture was heated under reflux connected to a Dean–Stark trap overnight. After 16 h more MeOH (3 × 15 mL) and *p*-TsOH·H_2_O (2 × 50 mg) were added and refluxing continued for 48 h. After cooling to rt the solvent was concentrated to about half its original volume and washed with 5% aq. NaOAc and brine. The organic phase was dried and evaporated to a light brown oil (0.96 g). Flash chromatography [EtOAc–hexanes (1 : 4) to (2 : 3)] gave two fractions. The first eluted material was unreacted starting material **2** (113 mg, 12%). The second eluted material was the lactone **4** (504 mg, 61%) as a viscous colourless oil which crystallised to white solid upon trituration with Et_2_O–hexanes, mp 110–111 °C (Et_2_O–hexanes). Anal. Calcd for C_21_H_20_NFO_5_: C, 65.45; H, 5.23; N, 3.63. Found: C, 65.39; H, 5.05; N, 3.6%. ^1^H NMR (800 MHz): *δ* 7.78 (d, *J* = 7.5 Hz, 2H, H_Ar_), 7.59 (t, *J* = 6.2 Hz, 2H, H_Ar_), 7.42 (t, *J* = 7.4 Hz, 2H, H_Ar_), 7.34 (t, *J* = 7.4 Hz, 2H, H_Ar_), 5.75 (d, *J* = 8.0 Hz, 1H, N*H*), 5.11 (dd, *J*_H–F_ = 47.0 Hz, *J*_H–H_ = 16.4 Hz, C*H*HF), 5.00 (dd, *J*_H–F_ = 47.0 Hz, *J*_H–H_ = 16.4 Hz, CH*H*F), 4.66–4.69 (m, 1H, C*H*–NH), 4.56 [dd, *J* = 10.6, 6.7 Hz, 1H, C*H*H(Fmoc)], 4.46 [dd, *J* = 10.6, 6.7 Hz, 1H, CH*H*(Fmoc)], 4.22 [t, *J* = 6.5 Hz, 1H, C*H*(Fmoc)], 3.70 (s, 3H, OC*H*_3_), 3.08 (dd, *J* = 17.3, 4.3 Hz, 1H, 4-H), 2.87 (dd, *J* = 17.3, 4.3 Hz, 1H, 4-H). ^13^C NMR (201 MHz): *δ* 202.67 (d, *J*_C–F_ = 17.1 Hz, 2-C), 171.58 (5-C), 155.87 [C=O(Fmoc)], 143.59 and 143.50 (C_Ar_), 141.41 and 141.39 (C_Ar_), 127.87 and 127.85 (*C*H_Ar_), 127.13 and 127.11 (*C*H_Ar_), 124.99 and 124.89 (*C*H_Ar_), 120.09 and 120.08 (*C*H_Ar_), 84.13 (d, *J*_C–F_ = 183.7 Hz, *C*H_2_F), 67.09 [*C*H_2_(Fmoc)], 54.05 (3-C), 52.30 (O*C*H_3_), 47.25 [*C*H(Fmoc)], 35.33 (C-4). ^19^F NMR (376 MHz): *δ* −231.79 [t (overlapped dd), *J*_H–F_ = 47.7 Hz]. The structure of the product was also confirmed by ^1^H**–**^1^H COSY and ^1^H–^13^C HSQC spectra (see ESI†).

#### (*S*)-3-{[(9*H*-Fluoren-9-yl)methoxycarbonyl]amino}-5-fluoro-4-oxopentanoic acid **5**

To a solution of **3** (748 mg, 1.75 mmol) in CH_2_Cl_2_ (50 mL), cooled to 0 °C was added TFA (10 mL) and the mixture was stirred at 0 °C for 0.5 h. The solution was then concentrated, and the residue was flash chromatographed [EtOAc–hexanes (1 : 4) to (3 : 2)] to give **5** (581 mg, 89%) as white foam-solid. ^1^H NMR (400 MHz): *δ* 7.77 (d, *J* = 7.3 Hz, 2H, H_Ar_), 7.59 (d, *J* = 7.3 Hz, 2H, H_Ar_), 7.39 (t, *J* = 7.4 Hz, 2H, H_Ar_), 7.34 (dt, *J* = 7.8, 0.9 Hz, 2H, H_Ar_), 5.33 (br s, 1H, N*H*), 4.32–4.70 [m, 5H, C*H*_2_F, C*H*–NH, C*H*_2_(Fmoc)], 4.19 [t, *J* = 6.4 Hz, C*H*(Fmoc)], 2.85–3.12 (m, 1H, half C*H*_2_CO_2_H), 2.58–2.77 (m, 1H, half C*H*_2_CO_2_H). ^19^F NMR (376 MHz): *δ* −231.85 [t (overlapped dd), *J*_H–F_ = 47.7 Hz].

#### Methyl (*S*)-3-{[(9*H*-fluoren-9-yl)methoxycarbonyl]amino}-5-fluoro-4-oxopentanoate **6**

To a solution of **3** (855 mg, 2 mmol) in CH_2_Cl_2_ (20 mL), cooled to 0 °C was added TFA (5 mL) and the mixture was stirred at 0 °C for 0.5 h. The solution was then concentrated, and the residue was re-evaporated from chloroform and dried under high vacuum. The residue was then dissolved in MeOH (25 mL) and treated with Me_3_SiCl (334 mg, 4 mmol) and stirred at rt overnight. The solution was concentrated, and the residue was flash chromatographed [EtOAc–hexanes (3 : 7)] to give **6** (456 mg, 59%) as a viscous colourless oil which crystallised to a white solid upon trituration with Et_2_O–hexanes, mp 106–107 °C (EtOAc–hexanes). Anal. Calcd for C_21_H_20_NFO_5_: C, 65.45; H, 5.23; N, 3.63. Found: C, 65.47; H, 5.34; N, 3.72%. ^1^H NMR (600 MHz): *δ* 7.80 (d, *J* = 7.6 Hz, 2H, H_Ar_), 7.55–7.63 (m, 2H, H_Ar_), 7.44 (t, *J* = 7.4 Hz, 2H, H_Ar_), 7.35 (t, *J* = 7.4 Hz, 2H, H_Ar_), 5.74 (d, *J* = 8.2 Hz, 1H, N*H*), 5.13 (dd, *J*_H–F_ = 47.3 Hz, *J*_H–H_ = 16.4 Hz, C*H*HF), 5.01 (dd, *J*_H–F_ = 47.0 Hz, *J*_H–H_ = 16.4 Hz, CH*H*F), 4.67–4.72 (m, 1H, C*H*–NH), 4.58 [dd, *J* = 10.6, 6.6 Hz, 1H, C*H*H(Fmoc)], 4.47 [dd, *J* = 10.6, 6.7 Hz, 1H, CH*H*(Fmoc)], 4.24 [t, *J* = 6.5 Hz, C*H*(Fmoc)], 3.72 (s, 3H, OC*H*_3_), 3.11 (dd, *J* = 17.3, 4.3 Hz, 1H, C*H*HCO_2_Me), 2.88 (dd, *J* = 17.2, 4.3 Hz, 1H, CH*H*CO_2_Me). ^19^F NMR (376 MHz): *δ* −232.00 and 231.74 [t, *J*_H–F_ = 47.1 Hz, Hz, rotamers (9 : 1)].

#### (*S*)-3-{[(9*H*-Fluoren-9-yl)methoxycarbonyl]amino}-5-bromo-4-oxopentanoic acid **7**

To a solution **2** (635 mg, 1.3 mmol) in CH_2_Cl_2_ (40 mL), cooled to 0 °C was added TFA (10 mL) and the mixture was stirred at 0 °C for 0.5 h. The solution was then concentrated, and the residue was flash chromatographed [EtOAc–hexanes (1 : 4) to (4 : 1)] to give **7** (534 mg, 95%) as pale solid. ^1^H NMR (400 MHz): *δ* (t, *J* = 7.3 Hz, 2H, H_Ar_), 7.31 (t, *J* = 7.3, Hz, 2H, H_Ar_), 5.74 [br s, 1H, N*H*], 4.50–4.80 [m, 5H, C*H*_2_F, C*H*–NH, C*H*_2_(Fmoc)], 4.419 [t, *J* = 6.4 Hz, C*H*(Fmoc)], 2.85–3.10 (m, 1H, half C*H*_2_CO_2_H), 2.55–3.10 (m, 1H, half C*H*_2_CO_2_H).

#### Methyl (*S*)-3-{[(9*H*-fluoren-9-yl)methoxycarbonyl]amino}-5-bromo-4-oxopentanoate **8**

To a solution of **2** (1.46 g, 3 mmol) in CH_2_Cl_2_ (30 mL), cooled to 0 °C was added TFA (7.55 mL) and the mixture was stirred at 0 °C for 1 h. The solution was then concentrated, and the residue was re-evaporated from chloroform and dried under high vacuum. The residue was then dissolved in MeOH (30 mL) and treated with Me_3_SiCl (0.65 g, 6 mmol) and stirred at rt overnight. The solution was concentrated, and the residue was flash chromatographed [EtOAc–hexanes (3 : 7)] to give **8** (1.04 g, 78%) as a viscous colourless oil which crystallised to white solid upon trituration with Et_2_O–hexanes, mp 138–140 °C (EtOAc–hexanes). ^1^H NMR (950 MHz): *δ* 7.79 (d, *J* = 7.5 Hz, 2H, H_Ar_), 7.60 (t, *J* = 6.7 Hz, 2H, H_Ar_), 7.43 (t, *J* = 7.3 Hz, 2H, H_Ar_), 7.34 (t, *J* = 7.3 Hz, 2H, H_Ar_), 5.78 [d, *J* = 8.5 Hz, 1H, N*H*], 4.74 (dt, *J* = 4.9, 4.3 Hz, 1H, C*H*–NH), 4.61 [dd, *J* = 10.8, 6.6 Hz, 1H, C*H*H(Fmoc)], 4.48 [dd, *J* = 10.8, 6.6 Hz, 1H, CH*H*(Fmoc)], 4.23 [t, *J* = 6.5 Hz, C*H*(Fmoc)], 4.07 (s, 2H, C*H*_2_Br), 3.71 (s, 3H, OC*H*_3_), 3.05 (dd, *J* = 17.4, 4.8 Hz, 1H, C*H*HCO_2_Me), 2.83 (dd, *J* = 17.4, 4.8 Hz, 1H, CH*H*CO_2_Me). ^13^C NMR (101 MHz): *δ* 199.8 (C_q_, C=O), 171.8 (C_q_, C=O), 155.9 (C_q_, C=O), 143.5 (C_q_, C_Ar_), 141.4 (C_q_, C_Ar_), 127.9 (CH, C_Ar_), 127.1 (CH, C_Ar_), 124.9 (CH, C_Ar_), 120.1 (CH, C_Ar_), 67.0 (CH_2_Br), 54.5 (CH) 52.3 (CH_3_), 47.3 (CH), 35.5 (CH_2_), 32.3 (CH_2_). Anal. Calcd for C_21_H_20_NBrO_5_: C, 56.52; H, 4.52; N, 3.14. Found: C, 56.64; H, 4.59; N, 3.16.

#### (9*H*-Fluoren-9-yl)methyl [(3*S*)-2-(bromomethyl)-2-methoxy-5-oxotetrahydrofuran-3-yl]-carbamate **9**

To a solution of **2** (0.73 g, 1.5 mmol) in MeOH (20 mL) and 2,2-dimethoxypropane (20 mL) was added *p*-TsOH·H_2_O (291 mg, 0.15 mmol) and the mixture was heated to 60 °C under reflux overnight. After cooling to rt the solvent was evaporated, and the residue diluted with EtOAc (50 mL) and washed with 5% aq. NaOAc and brine. The organic phase was dried and evaporated to a light brown oil (0.86 g). Flash chromatography [EtOAc–hexanes (1 : 4) to (1 : 3)] gave two fractions. The first eluted material was unreacted starting material **2** (391 mg, 53%). The second eluted material was the cyclic product **9** (123 mg, 18%) as white crystals, mp 140–142 °C (Et_2_O–hexanes). ^1^H NMR (400 MHz): *δ* 7.76 (d, *J* = 7.5 Hz, 2H, H_Ar_), 7.58 (dd, *J* = 7.6, 2.8 Hz, 2H, H_Ar_), 7.41 (t, *J* = 7.5 Hz, 2H, H_Ar_), 7.32 (t, *J* = 7.4 Hz, 2H, H_Ar_), 5.82 (d, *J* = 8.8 Hz, 1H, N*H*), 4.72 (dt, *J* = 9.2, 4.9 Hz, 1H, C*H*–NH), 4.58 [dd, *J* = 10.8, 6.5 Hz, 1H, C*H*H(Fmoc)], 4.46 [dd, *J* = 10.8, 6.5 Hz, 1H, CH*H*(Fmoc)], 4.20 [t, *J* = 6.3 Hz, 1H, C*H*(Fmoc)], 4.05 (s, 2H, *CH*_2_Br), 3.68 (s, 3H, OC*H*_3_), 3.02 (dd, *J* = 17.4, 5.0 Hz, 1H, 4-H), 2.81 (dd, *J* = 17.4, 4.9 Hz, 1H, 4H). ^13^C NMR (101 MHz): *δ* 199.6 (C_q_, C=O), 171.8 (C_q_, C=O), 155.9, (C_q_), 143.5 (C_q_, C_Ar_), 141.4 (C_q_, C_Ar_), 127.9 (CH, C_Ar_), 127.1 (CH, C_Ar_), 124.9 (CH, C_Ar_), 120.1 (CH, C_Ar_), 67.0 (CH_2_, CH_2_Br), 54.5 (CH), 52.3 (CH_3_), 47.3 (CH), 35.5 (CH_2_), 32.3 (CH_2_).

#### Methyl (*S*)-3-{[(9*H*-fluoren-9-yl)methoxycarbonyl]amino}-5-bromo-4-oxopentanoate **8**

To a solution of Fmoc-l-Asp(OMe)-OH (7.39 g, 20 mmol) in dry THF (100 mL), cooled to −10 °C was added *N*-ethylmorpholine (NEM) (2.88 g, 25 mmol) and isobutyl chloroformate (3.14 g, 25 mmol). A white precipitate was formed instantly, and the mixture was stirred at −10 °C for 1 h. Diazomethane, generated *in situ* using the procedure described in Aldrich Technical Bulletin (AL-180) (66.4–85.6 mmol) in Et_2_O, dried over KOH pellets, was added slowly to the reaction mixture at 0 °C and the mixture was allowed to warm to rt overnight with stirring. The mixture was then cooled to 0 °C and treated with a solution of 33% HBr in AcOH and water (1 : 2) (60 mL) and stirred at 0 °C for 1 h and the at rt for further 1 h until the evolution of gas ceased. The mixture was diluted with EtOAc (200 mL) and washed sequentially with water, brine and saturated aq. NaHCO_3_. The organic phase was dried and evaporated to a pale viscous oil which crystallised after trituration with ether. Recrystallization afforded **8** (7.83 g, 88%) as white crystals, mp 138–140 °C (EtOAc–hexanes) identical to the compound described above.

#### Methyl (*S*)-3-{[(9*H*-fluoren-9-yl)methoxycarbonyl]amino}-5-fluoro-4-oxopentanoate **6**

To a solution of **8** (2.23 g, 5 mmol) and *p*-TsOH·H_2_O (2.87 g, 15 mmol) in THF (750 mL) was added dropwise a solution of TBAF^.^H_2_O (7.84 g, 48 mmol) in THF (75 mL) and the mixture was stirred in a nitrogen atmosphere under reflux overnight. After cooling to rt the solvent was evaporated, and the residue was dissolved in EtOAc (150 mL) and washed with water and brine. The organic phase was dried and evaporated, and the residue was flash chromatographed [EtOAc–hexanes (1 : 4) to (3 : 7)] to give **6** (0.43 g, 22%) as pale crystals, mp 106–107 °C (EtOAc–hexanes) identical to the compound described above.

#### (1*s*,4*r*)-4-[(*S*)-11-(9*H*-Fluoren-9-yl)-6-(fluoromethyl)-7-(2-methoxy-2-oxoethyl)-3,9-dioxo-10-oxa-2,4,5,8-tetraazaundec-5-en-1-yl]cyclohexane-1-carboxylic acid **11**

To a solution of semicarbazidyl-*trans*-4-methylcyclohexane carboxylic acid trifluoroacetate salt **10** (342 mg, 1.04 mmol) in EtOH (20 mL) was added water (3 mL) and sodium acetate trihydrate (212 mg, 1.56 mmol). Fluoromethyl ketone **6** (401 mg, 1.04 mmol) was added and the mixture was heated under reflux in a nitrogen atmosphere for 3 h. After cooling to room temperature, the solution was diluted with EtOAc (80 mL) and washed with 0.1 M aq. HCl and brine, dried and evaporated a pale foam (587 mg, 97%) which was used in the next step without further purification. ^1^H NMR (400 MHz, MeOD): *δ* 7.77–7.83 (m, 2H), 7.62–7.67 (m, 2H), 7.40 (dtt, *J* = 7.7, 3.2, 1.3 Hz, 2H), 7.28–7.36 (m, 2H), 4.97–5.19 (m, 1H), 4.18–4.6262 (m, 4H), 3.76 (s, 1H), 3.61–3.70 (m, 2H), 2.96–3.19 (m, 1H), 2.63–3.01 (m, 2H), 2.36–2.47 (m, 1H), 2.13–2.30 (m, 1H), 1.92–2.08 (m, 2H), 1.84 (d, *J* = 15.4 Hz, 2H), 1.56–1.28 (m, 2H), 1.19–1.28 (m, 3H), 0.87–1.12 (m, 2H). MS (ES^+^) *m*/*z*: 582.0 [M + H]^+^; calcd for C_30_H_35_N_4_FO_7_: 582.2.

#### Preparation of resin **12**

Compound **11** (436 mg, 0.748 mmol 1.1 equiv.) was attached to resin-bound methylbenzhydrylamine (MBHA) hydrochloride (200–400 mesh, 0.88 mmol g^−1^ loading capacity) (773 mg, 0.68 mmol) in DMF (3 mL) using HATU (272 mg, 0.714 mmol, 1.05 equiv.) and DIPEA (1.185 mL, 6.8 mmol, 10 equiv.) at rt for 16 h. The resin was washed with DMF and used in further steps.

#### Synthesis of Z-VAD(OMe)-FMK **13**

This peptide was synthesised manually by coupling resin **12** (0.14 mmol) scale with Fmoc-Ala-OH followed by Z-Val-OH using standard solid phase Fmoc procedures. Prior to each coupling the Fmoc was removed by treating the resin with (3 × 10 mL) of 20% piperidine in DMF for 10 min. HATU (4.5 equiv.) and DIPEA (10 equiv.) were used as the coupling reagents with 5-fold excess of each amino acid. Cleavage was performed by treatment of the peptide-resin with an ice-cold solution of (95% TFA, 2.5% H_2_O, 2.5% TIPS) for 30 min. Following filtration to remove the linker retaining resin, ether was added to the filtrate. The peptide was ether soluble so following evaporation of ether the peptide was solubilised in water and acetonitrile prior to lyophilisation. Aliquots of the peptide were solubilised in a (2 : 1 : 1) mixture of H_2_O–MeCN–MeOH (2 mL) and purified on a C8 reverse phase HPLC column (Agilent PrepHT Zorbax 300SB-C8, 21.2 × 250 mm, 7 m) using a linear solvent gradient of 15–60% (MeCN + 0.08% TFA) and (H_2_O + 0.08% TFA) over 40 min at a flow rate of 8 mL min^−1^. Fractions containing the product were combined and lyophilised to dryness to give peptide **13** (4.4 mg, 6.7%). The purified peptide was analysed by LCMS on an Agilent 1100 LC-MSD; estimated purity 65%; MS (ES^+^) *m*/*z*: 468.0 [M + H]^+^, 489.9 [M + Na]^+^; calcd for C_22_H_30_N_3_FO_7_: 467.2.

#### SPPS of Z-AVLD(OMe)-FMK **14**, Z-SAVLD(OMe)-FMK **15** and Z-ASAVLD-FMK **16**

The peptides were synthesised on an Intavis ResPep SLi automated synthesiser (Intavis Bioanalytical Instruments AG, Cologne Germany), on a 0.1 mmol scale using Fmoc-amino acids, Z-amino acids, (5 equiv.), HATU (5 equiv.) and DIPEA (10 equiv.) with respect to resin **12**. The peptides were cleaved from the resin by treatment with of an ice-cold cleavage solution (95% TFA, 2.5% H_2_O, 2.5% TIPS) for 30 min at 0 °C. Following filtration to remove the linker retaining-resin, ether was added to the filtrate. The peptides were ether soluble so following evaporation of ether the individual peptide was solubilised in water and acetonitrile prior to lyophilisation. Each peptide was solubilised in MeOH (1 mL) and purified on a C8 reverse phase HPLC column (Agilent PrepHT Zorbax 300SB-C8, 21.2 × 250 mm, 7 m) using a linear solvent gradient of 15–65% (MeCN + 0.08% TFA) and (H_2_O + 0.08% TFA) over 40 min at a flow rate of 8 mL min^−1^. Fractions containing the product were pooled and lyophilised to dryness and analysed by LCMS on an Agilent 1100 LC-MSD (see ESI†).

Z-AVLD(OMe)-FMK **14** (1.4 mg, 2.4%); estimated purity 37%; MS (ES^+^) *m*/*z*: 581.3 [M + H]^+^, 603.3 [M + Na]^+^; calcd for C_28_H_41_N_4_FO_8_: 580.3;

Z-SAVLD(OMe)-FMK **15** (0.8 mg, 1.2%); estimated purity 65%; MS (ES^+^) *m*/*z*: 668.3 [M + H]^+^, 690.3 [M + Na]^+^; calcd for C_31_H_46_N_5_FO_10_: 667.3;

Z-ASAVLD(OMe)-FMK **16** (0.6 mg, 0.8%); estimated purity 33%; MS (ES^+^) *m*/*z*: 739.4 [M + H]^+^, 761.5 [M + Na]^+^; calcd for C_34_H_51_N_6_FO_11_: 738.4.

#### 3CL^pro^ gel-based protease assay

Reactions were carried out as described previously.^33^ Briefly, the proteolytic activity of 3CL^pro^ was assessed by monitoring the cleavage of a fusion protein substrate (FLAG-His-SAVLQ-nsp9) over time. In this instance, 1 μM of 3CL^pro^ in a reaction buffer containing 50 mM HEPES–KOH pH 7.6, 1 mM EDTA, 2 mM DTT, 10% glycerol, and 0.02% Tween-20 was incubated with either varying concentrations of Z-SAVLD(OMe)-FMK or DMSO. The cleavage reaction was then initiated by the addition of 6.25 μM of FLAG-His-SAVLQ-nsp9 for 1 hour at room temperature (19–23 °C). The reactions were then quenched and denatured by the addition of SDS loading buffer and separated over a 12% Bis-Tris gel in MOPS buffer and stained with coomassie blue (Generon Cat. No. NB-45-00078-1L).

## Conflicts of interest

There are no conflicts to declare.

## Supplementary Material

RA-011-D1RA03046A-s001
