# Supplementary material for: An improved method for the incorporation of fluoromethyl ketones into solid phase peptide synthesis techniques
Source: RSC Adv. 2021 Jun 8;11(33):20457–64. doi: 10.1039/d1ra03046a (PMC8185805; doi:10.1039/d1ra03046a)

Electronic Supplementary Material (ESI) for RSC Advances.
This journal is © The Royal Society of Chemistry 2021

Supporting information for

An improved method for the incorporation of fluoromethyl ketones into solid phase peptide synthesis techniques

by

[a] Dhira Joshi,[a] Jennifer C. Milligan,[b] Theresa U. Zeisner,[c] Nicola O'Reilly,[a] and John F.X. Diffley[b] and George Papageorgiou[a]*
**Corresponding author*

E-mail: George.Papageorgiou@crick.ac.uk, Tel: (+) 44 203 796 2359
[a]Peptide Chemistry STP
[b]Chromosome Replication Laboratory
[c] Cell Cycle Laboratory
The Francis Crick Institute, 1 Midland Road, London NW1 1AT

Contents

Schematic presentation for synthesis of linker **10** S2
Benzyl (1*R*,4*R*)-4-(aminomethyl)cyclohexane-1-carboxylate *p*-tosylate (**S1**) S2
tert-Butyl 2-((((1R,4R)-4((benzyloxy)carbonyl)cyclohexyl)methyl)carbamoyl)hydrazine-1-carboxylate (**S2**) S2
(1*R*,4*R*)-4-((2-(*tert*-Butoxycarbonyl)hydrazine-1-carboxamido)methyl)cyclohexane-1-carboxylic acid (**S3**) S2
(1*R*,4*R*)-4-(Hydrazinecarboxamidomethyl)cyclohexane-1-carboxylic acid trifluoroacetate **10** S3
^{1}H-^{1}H-COSY spectrum of compound **4** S3
^{1}H-^{13}C-HSQC spectrum of compound **4** S4
Chromatogram of compound **11** S4
Chromatogram of Z-VAD(OMe)-FMK **13** S5
LCMS of Z-VAD(OMe)-FMK **13** S5
Chromatogram of Z-AVLD(OMe)-FMK **14** S6
LCMS of Z-AVLD(OMe)-FMK **14** S6
Chromatogram of Z-SAVLD(OMe)-FMK **15** S7
LCMS of Z-SAVLD(OMe)-FMK **15** S7
Chromatogram of Z-ASAVLD(OMe)-FMK **16** S8
LCMS of Z-ASAVLD(OMe)-FMK **16** S8

Schematic presentation for synthesis of linker 10.

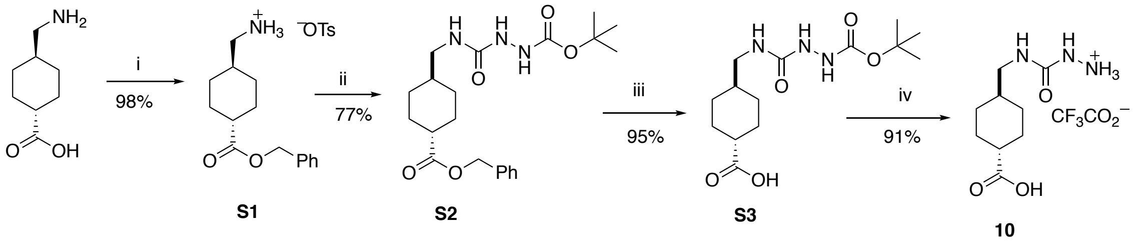

Scheme 1. Reagents: (i) $PhCH_2OH$, *p*-TsOH, Toluene, reflux; (ii) *t*-butyl carbazate, CDI, DMF, Et_3N; (iii) MeOH, H_2, 10% Pd-C; (iv) TFA, 0 ˚C

Benzyl (1*R*,4*R*)-4-(aminomethyl)cyclohexane-1-carboxylate *p*-tosylate S1

A solution of *trans*-4-(aminomethyl)cyclohexanecarboxylic acid (tranexamic acid) (10.0 g, 63.6 mmol) and *p*-toluenesulfonic acid monohydrate (12.34 g, 64.9 moles) in a mixture of benzyl alcohol (50 mL) and toluene (50 mL) was heated to reflux for 24 h The liberated water was azeotropically removed with Dean-Stark trap. The clear solution obtained was allowed to cool to rt and the product crystallized out. The solid was filtered, washed with ether and dried in a vacuum desiccator to give **S1** (26.01 g, 98%) as a white solid, mp 155-157 ˚C, Lit.[1] mp 154-156°C.

tert-Butyl 2-((((1R,4R)-4((benzyloxy)carbonyl)cyclohexyl)methyl)carbamoyl)hydrazine-1-carboxylate S2

To a solution of carbonyldiimidazole (CDI) (9.73 g, 60 mmol) in dry DMF (135 mL) was added dropwise a solution of *t*-butyl carbazate (7.93 g, 60 mmol) in dry DMF (135 mL) over 30 min under a nitrogen atmosphere. The resulting mixture was treated portionwise with *trans*-4-(aminomethyl)-cyclohexanecarboxylic acid benzyl ester para-toluenesulfonate salt **S1** (25 15 g, 60 mmol), followed by the dropwise addition of triethylamine (9 ml) over a period of 30 min. The reaction mixture was allowed to stir at rt under nitrogen for 2 h. Water (300 mL) was added, and this mixture was washed with EtOAc (3 × 250 mL). The combined organic layers were washed with 1M aq. HCI, saturated aq. $NaHC0_3$ and brine, dried and evaporated to give **S2** (18.80 g 77%) as a pale solid. This material was used in the next step without further purification.

(1*R*,4*R*)-4-((2-(*tert*-Butoxycarbonyl)hydrazine-1-carboxamido)methyl)cyclohexane-1-carboxylic acid S3

To a solution of the crude Boc-benzyl ester **S2** (18.78 g, 46.3 mmol) in MeOH (150 mL) was added 10% palladium on activated carbon (1.25 g) and hydrogenated at atmospheric pressure for a few hours until about (1037 mL) of hydrogen was taken up. The solid was filtered off and the filtrate was concentrated to a foam. Dichloromethane was then added, and the mixture was kept at 5°C overnight. The crystallized material was filtered and washed with ether to give **S3** (8.27 g, 57%) as a white solid. This material was used in the next step without further purification.

(1*R*,4*R*)-4-(Hydrazinecarboxamidomethyl)cyclohexane-1-carboxylic acid trifluoroacetate 10

Compound **S3** (8.25 g, 26.2 mmol) was dissolved in TFA (62 mL) at 0°C and stirred for 30 min. The solution was concentrated to a small volume and dry Et_2O (125 mL) was added. The precipitate formed was filtered off, washed with cold ether and dried a vacuum desiccator to give **10** (7.87 g, 91%), as a white solid. A portion of the product (2.56 g) was chromatographed on Biotage Isolera One 3.3.0 loading on a isofar (50 g) column and eluting with [$CHCl_3$-MeOH (0 to 30%)] mp 155-157 ˚C, Lit.[1] mp 154-156°C. ^{1}H NMR (400 MHz, MeOD) δ 3.04 (d, *J* = 6.8 Hz, 2H), 2.21 (tt, *J* = 12.2, 3.4 Hz, 1H), 2.00 (dt, *J* = 13.7, 3.3 Hz, 2H), 1.93 – 1.78 (m, 2H), 1.55 – 1.31 (m, 3H), 1.02 (dqd, *J* = 25.2, 12.7, 3.5 Hz, 2H). ^{13}C NMR (101 MHz, MeOD) δ 179.8, 159.5, 47.0, 44.4, 38.98, 30.7, 29.8.

References

1 A. M. Murphy, R. Dagnino, P. L. Vallar, A. J. Trippe, S. L. Sherman, R. H. Lumpkin, S. Y. Tamura and T. R. Webb, *J. Am. Chem. Soc.*, 1992, **114**, 3156–3157.

^{1}H-^{1}H-COSY spectrum of compound 4

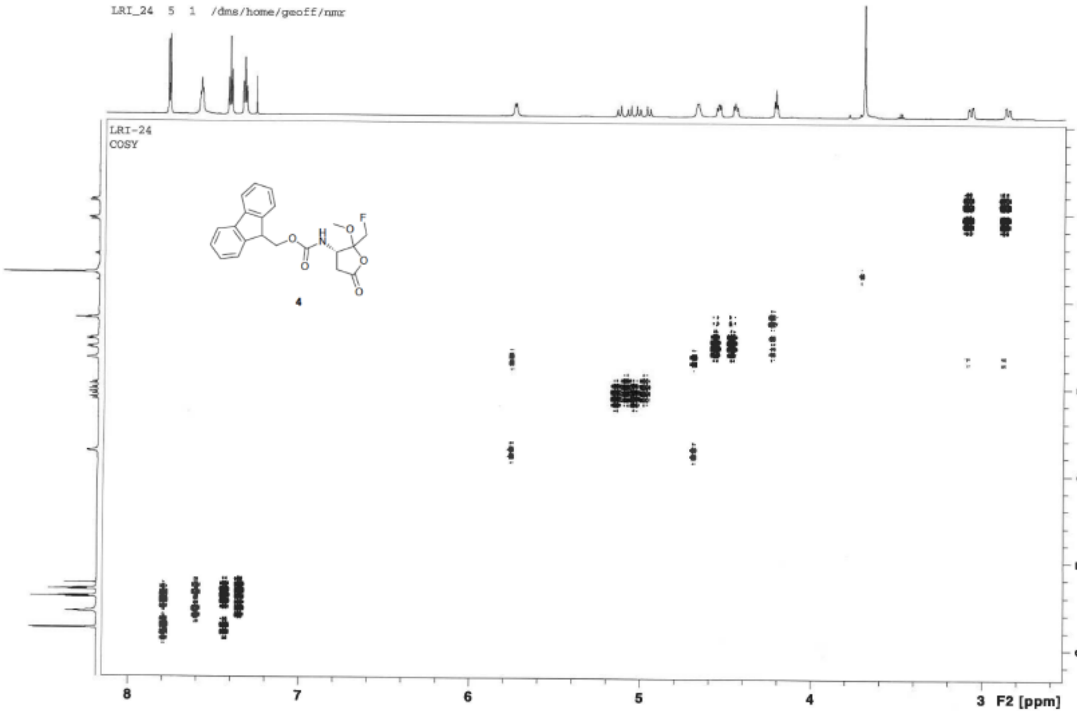

^{1}H-^{13}C-HSQC spectrum of compound 4

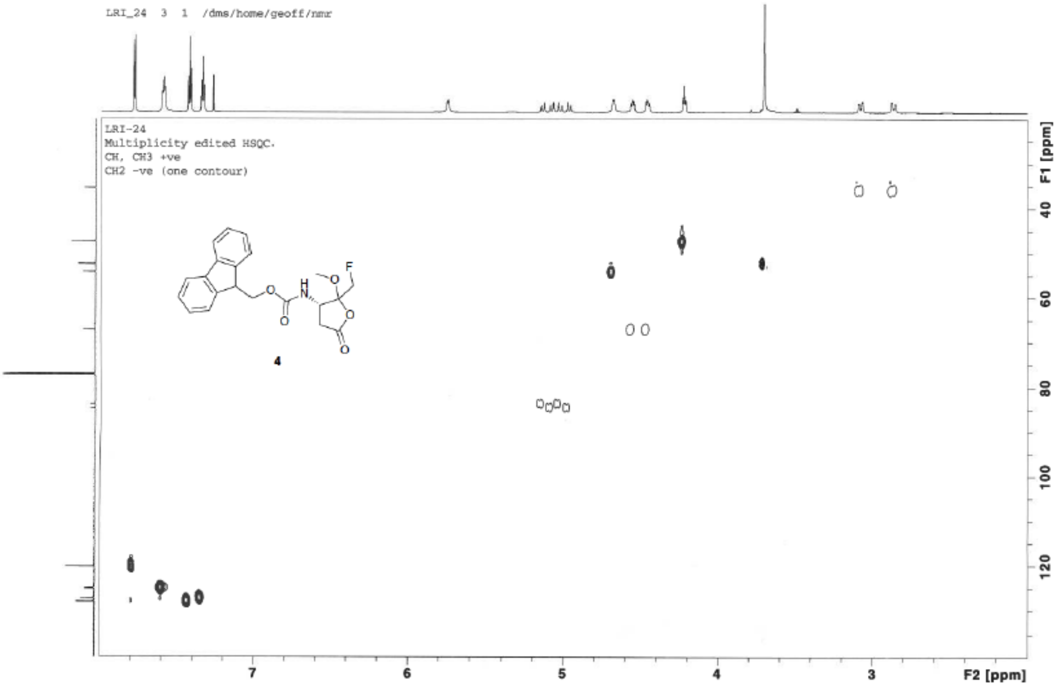

Chromatogram of compound 11

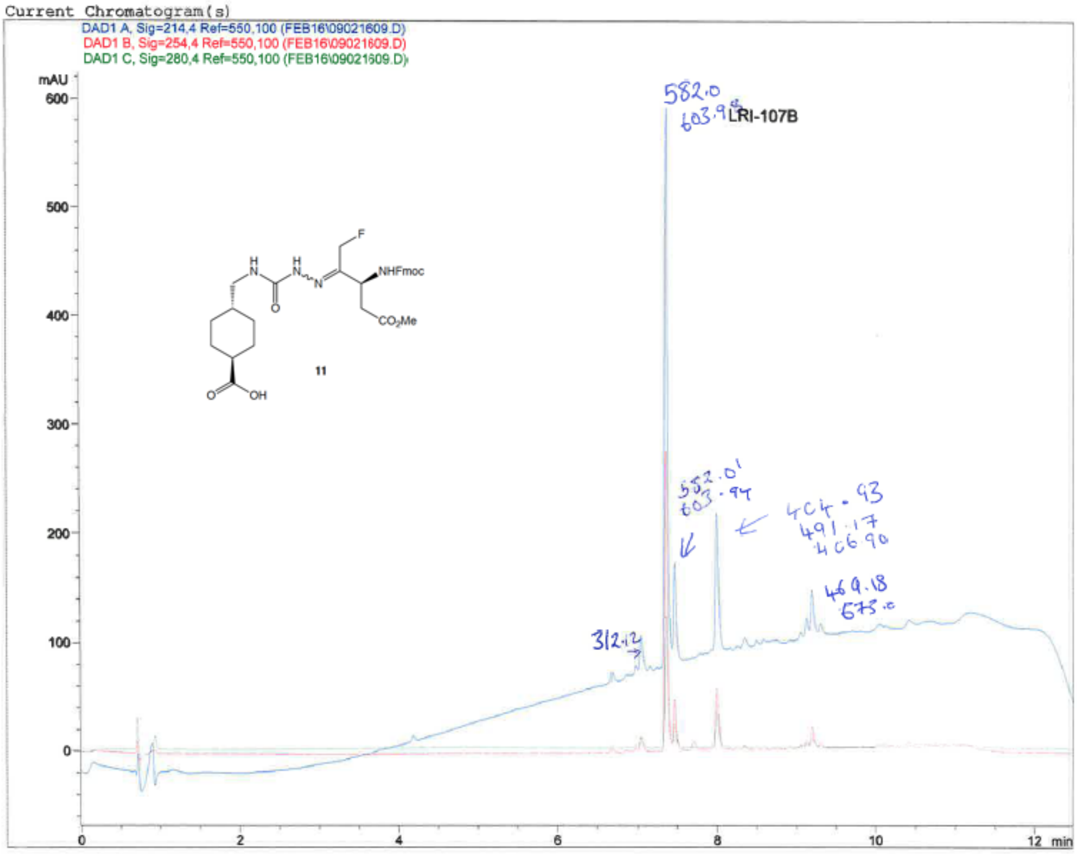

Chromatogram of Z-VAD(OMe)-FMK 13

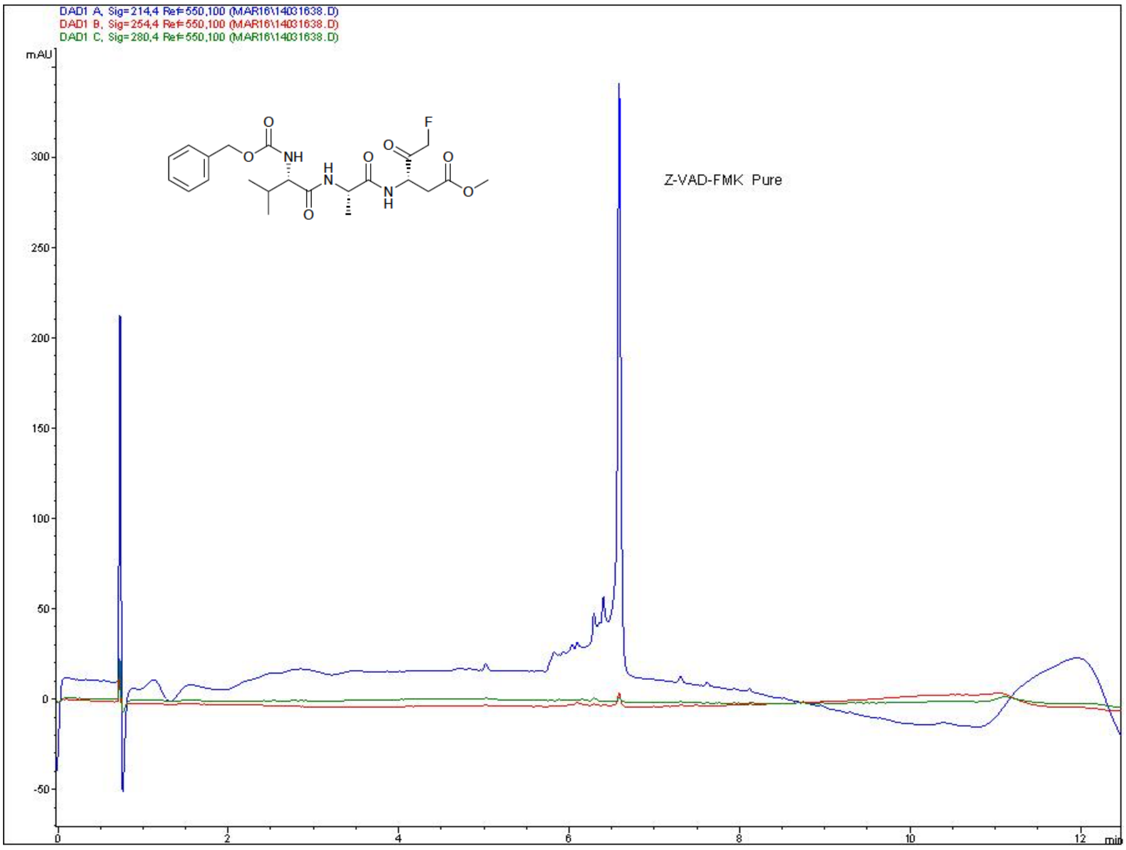

LCMS of Z-VAD(OMe)-FMK 13

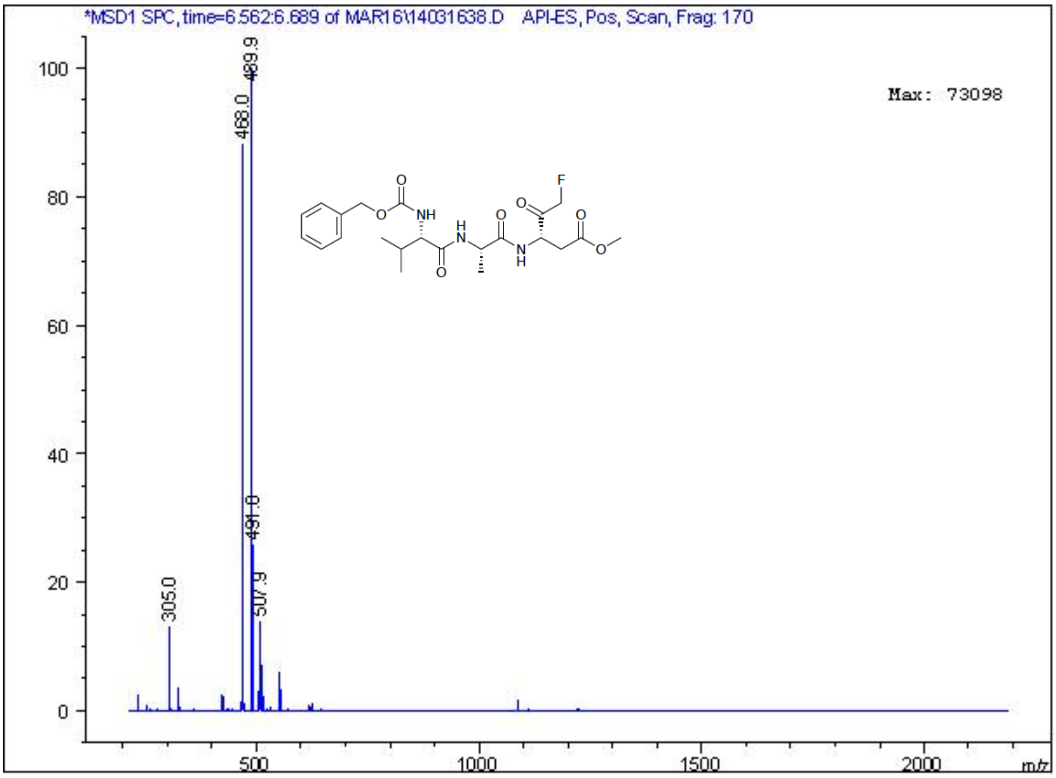

Chromatogram of Z-AVLD(OMe)-FMK 14

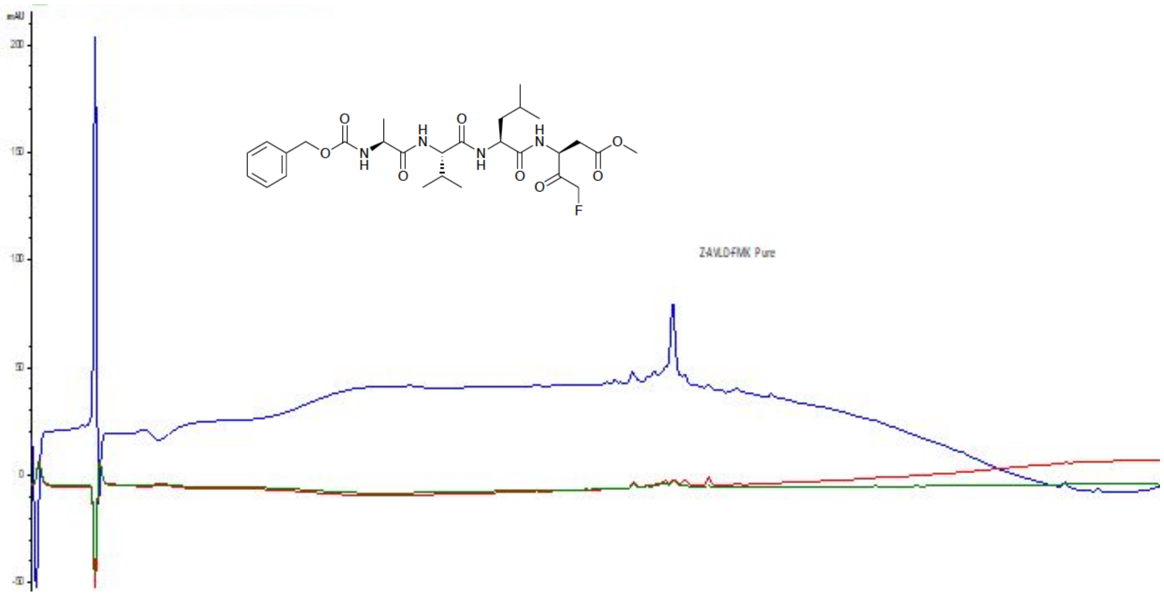

LCMS of Z-AVLD(OMe)-FMK 14

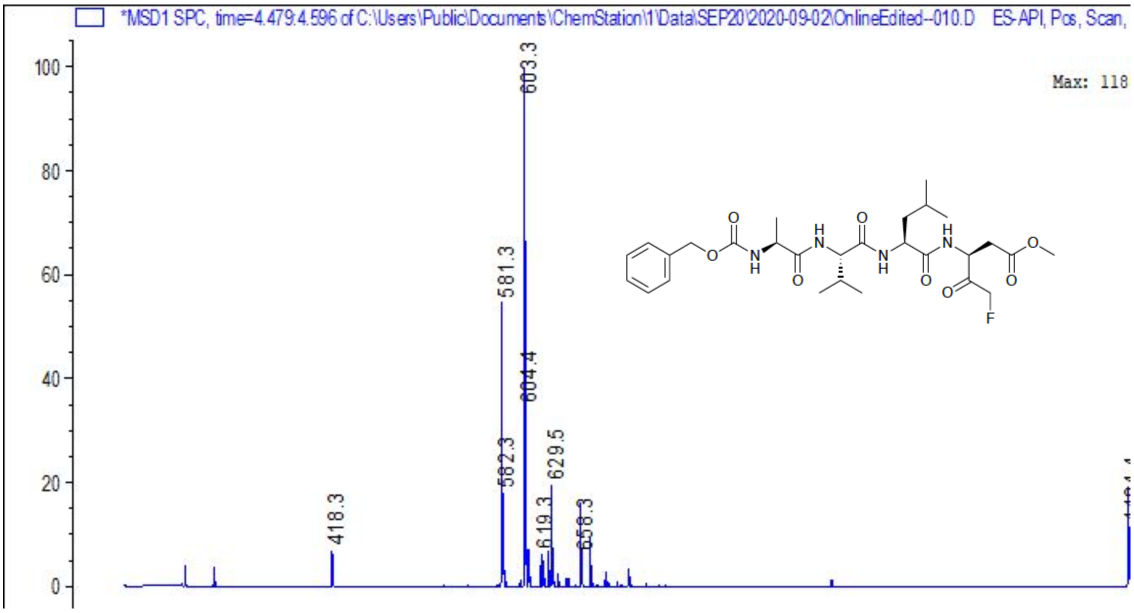

Chromatogram of Z-SAVLD(OMe)-FMK 15

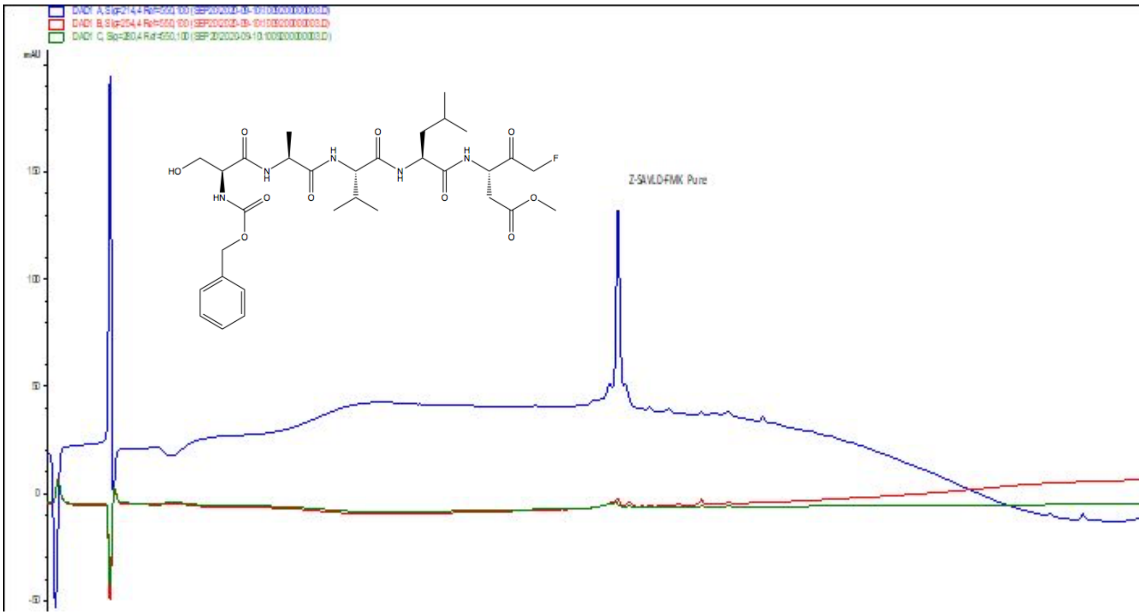

LCMS of Z-SAVLD(OMe)-FMK 15

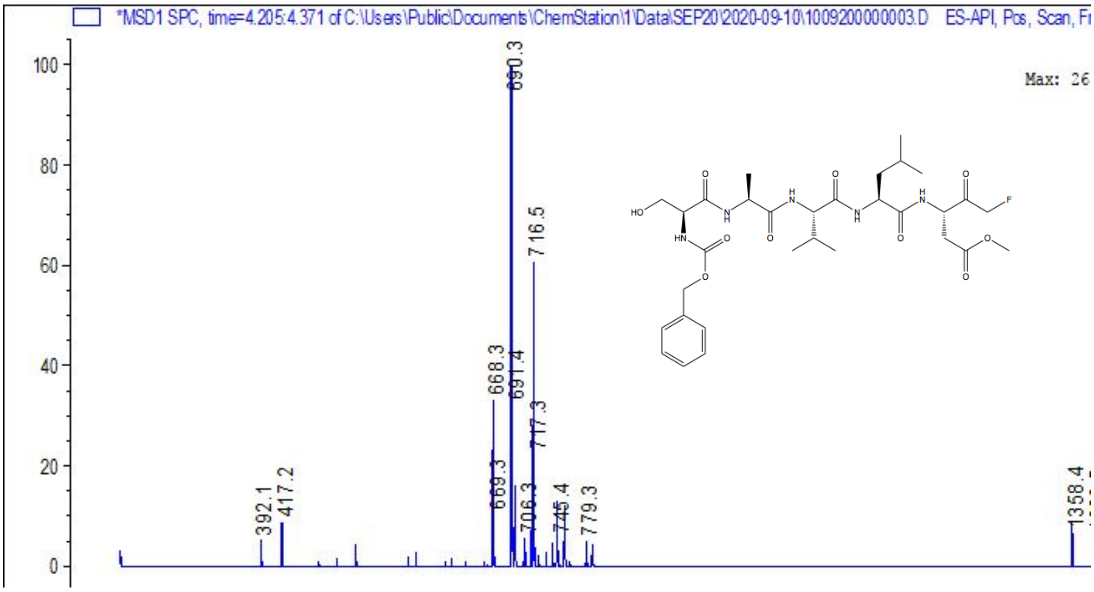

Chromatogram of Z-ASAVLD(OMe)-FMK 16

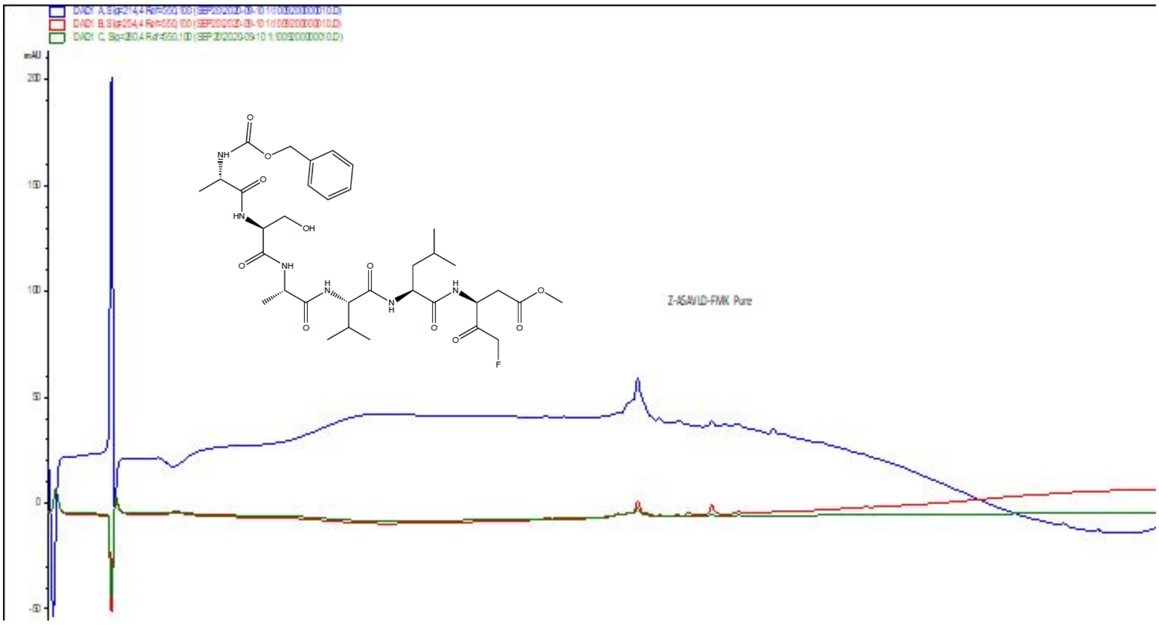

LCMS of Z-ASAVLD(OMe)-FMK 16

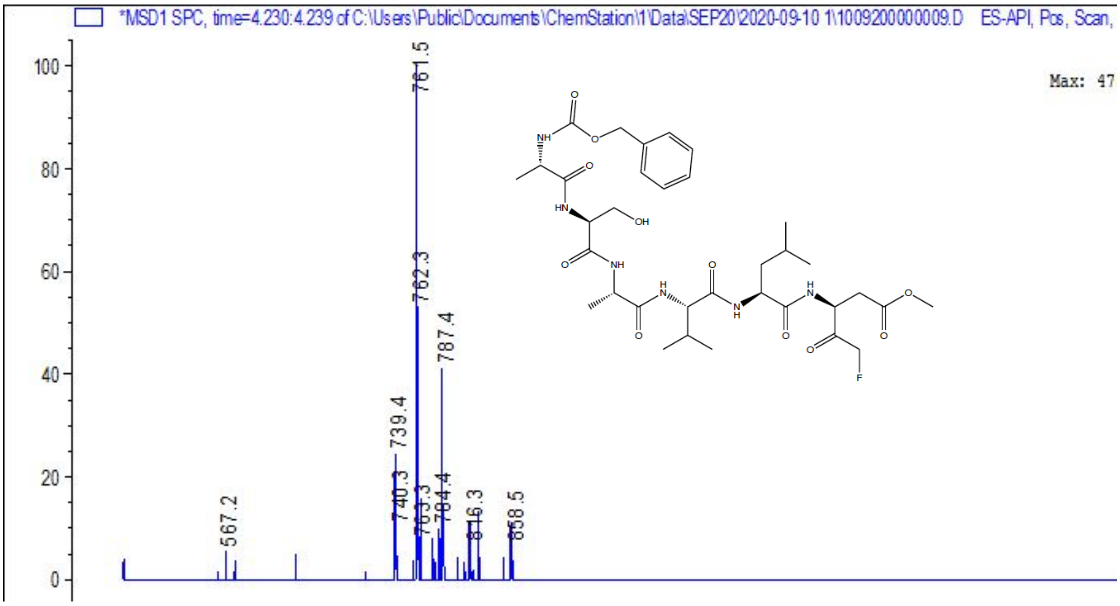

Supplement: RA-011-D1RA03046A-s001 [file RA-011-D1RA03046A-s001.pdf]
